# Activating and inhibiting connections in biological network dynamics

**DOI:** 10.1186/1745-6150-3-49

**Published:** 2008-12-04

**Authors:** Daniel McDonald, Laura Waterbury, Rob Knight, M D Betterton

**Affiliations:** 1 Department of Computer Science, University of Colorado, 430 UCB, Boulder, CO 80309, USA; 2 Department of Applied Mathematics, University of Colorado, 526 UCB, Boulder, CO 80309, USA; 3 Department of Chemistry and Biochemistry, University of Colorado, 215 UCB, Boulder, CO 80309, USA; 4 Department of Physics, University of Colorado, 390 UCB, Boulder, CO 80309, USA

## Abstract

**Background:**

Many studies of biochemical networks have analyzed network topology. Such work has suggested that specific types of network wiring may increase network robustness and therefore confer a selective advantage. However, knowledge of network topology does not allow one to predict network dynamical behavior – for example, whether deleting a protein from a signaling network would maintain the network's dynamical behavior, or induce oscillations or chaos.

**Results:**

Here we report that the balance between activating and inhibiting connections is important in determining whether network dynamics reach steady state or oscillate. We use a simple dynamical model of a network of interacting genes or proteins. Using the model, we study random networks, networks selected for robust dynamics, and examples of biological network topologies. The fraction of activating connections influences whether the network dynamics reach steady state or oscillate.

**Conclusion:**

The activating fraction may predispose a network to oscillate or reach steady state, and neutral evolution or selection of this parameter may affect the behavior of biological networks. This principle may unify the dynamics of a wide range of cellular networks.

**Reviewers:**

Reviewed by Sergei Maslov, Eugene Koonin, and Yu (Brandon) Xia (nominated by Mark Gerstein). For the full reviews, please go to the Reviewers' comments section.

## Background

Many biological processes involve networks of interacting proteins or genes. Examples include networks that control the cell cycle, transcriptional regulation, cellular signaling, and cell-fate determination in development. As more biochemical networks are mapped, detailed analysis of networks has become possible. Many researchers have analyzed the connections among nodes in the networks [1-3]. Different studies have emphasized the importance of network structure, motifs, or other properties [4-7]. While the topology of biochemical networks is informative – for example, feedback loops are necessary for oscillatory dynamics – topology does not fully describe network behavior. The dynamic response to different inputs is a key property that biological networks have evolved; perturbing the network can alter the dynamics [8], and the topological structure of the network may be a byproduct of selection for dynamical behavior [9-11]. Understanding the relationship between network topology and dynamics can give insight into the evolution of cellular oscillators and switches [12-14].

Using a simple model of biochemical network dynamics [15], we show that the balance between activating and inhibiting connections strongly influences whether network dynamics reach steady state or oscillate. A high fraction of activating connections predisposes network dynamics to reach steady state. Tuning this parameter alters the oscillation period. We found significant dependence of the dynamics on the fraction of activating connections in random networks, optimized networks, and examples of biological networks. Our work is related to previous work on the sign of interactions in transcription modules [16] and biological network subsystems [17].

**Figure 1:**
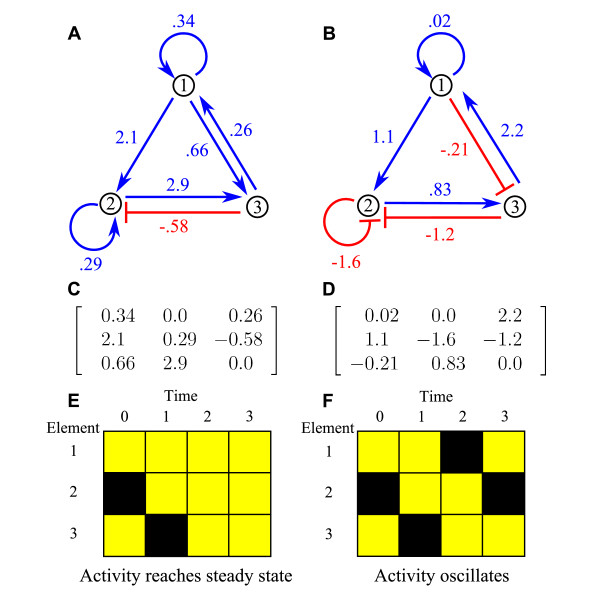
**Example networks and dynamics.** A and B: sketches of 3-node networks. Numbered points represent nodes and arrows interactions; blue/red arrows show activating/inhibiting connections. The interaction strength is given next to each arrow. The two networks have the same topology but differ in connection strengths and activating fraction of connections *a*; B shows a lower *a*. C and D: matrices *W *which represent networks A and B, where *W*_*ij *_is the interaction strength from node *j *to node *i*. E and F: Network dynamics with initial condition **s**(*t *= 0) = (1, -1, 1). Active/inactive nodes are represented by yellow/black squares The activity of network A reaches steady state after 2 iterations (E). The activity of network B undergoes period-3 oscillation (F).

Our model includes key features of a biochemical network: interactions of varying strengths, strongly nonlinear dynamics, and saturating response to inputs [15]. Variants of the model have been used to study robustness in genetic networks, with a focus on dynamics that reach steady state [18-21]. The model describes interactions among the nodes – which represent the genes, mRNA transcripts, or proteins – and the activity of each node – which represents the expression and/or activity level of the molecule. (Activity of a molecule changes if its concentration changes, or because of chemical changes such as phosphorylation.) The interaction strengths are given by the matrix *W*, where *W*_*ij *_is the strength of the effect of node *j *on node *i *(fig. 1). Each *W*_*ij *_can be positive (activating), negative (inhibiting), or zero (no interaction). The activating fraction *a *is the fraction of nonzero interaction strengths which are positive. Nodes can self-regulate, an effect known to be important in biochemical networks [22,23]. The activity vector is **s**, with *s*_*i *_the activity of node *i*. Each *s*_*i *_is between -1 ("off") and 1 ("on"), where 0 corresponds to the basal activity of the node. This model is similar to a class of models of neuronal networks [24], where the balance of activating and inhibiting connections is also of interest [25-27].

The activity vector changes in time according to

(1) **s**(*t *+ 1) = tanh(*rW***s**(*t*)).

The hyperbolic tangent saturates the interactions among nodes so that *s*_*i *_∈ [-1, 1]. We use *r *= 100 unless otherwise specified. The dynamics can reach steady state (fig. 1e), oscillate (fig. 1f) or change chaotically. The dynamics are fully specified by eq. 1 and the initial condition. We randomly assigned *s*_*i*_(0) = 1 or -1 with equal probability. We then determined whether the dynamics reach a steady state and the period of oscillation.

## Results and discussion

We first studied the dynamics of random Erdös-Renyi networks. We generated networks with specified size *N*, probability of nonzero connection between nodes *c*, activating fraction connections *a*, and random Gaussian *W*_*ij*_.

**Figure 2:**
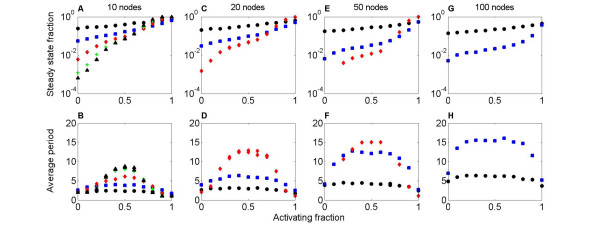
**The activating fraction and dynamics of random (Erdös-Renyi) networks.** A, C, E, G: Fraction of networks whose dynamics reach steady state, as a function of activating fraction *a*. B, D, F, H: Average oscillation period of network dynamics, as a function of *a*. The network size is 10 (A, B), 20 (C, D), 50 (E, F), or 100 (G, H). The number of connections per node is 1 (black circle), 2 (blue square), 5 (red diamond) 8 (green +), or 10 (black triangle). For *a *near 1, the network dynamics are highly likely to reach steady state, independent of other parameters (A, C, E, G). For *a *near 0, the steady state fraction is between 7 × 10^-4 ^and 0.25, depending on the network degree. For *a *near 1, the average period is close 1, which corresponds to steady state (B, D, F, H). For *a *near 0, the period is close to 2, because the activity of network nodes oscillates between off and on. For *a *near 0.5, the period has a maximum. The maximum oscillation period increases with the size and degree of the network.

For a wide range of conditions, the activating fraction *a *is strongly correlated with the probability that the dynamics of a random network reach steady state. For *a *near 1, nearly all runs of the network dynamics reach steady state, while for *a *near 0, few runs reach steady state; the network dynamics typically oscillate (fig. 2). When *a *= 0, ~0.01% to 10% of runs reach steady state, depending on network degree. The probability of reaching steady state is only weakly dependent on the size of the network, if the number of connections per node is fixed (fig. 2). Altering the typical magnitude of connection strengths has little effect on the dynamics, because the dynamics are highly saturated (see Methods).

The average period of oscillation varies strongly with *a*. When *a *= 1, most runs reach steady state (period 1), while for *a *= 0, period-2 oscillations are typical (the activity of each node switches between -1 and 1). The longest average period occurs for *a *= 0.5, where activating and inhibiting connections are equally likely (fig. 2). Increasing *N *or *c *tends to increase the period.

In random Erdös-Renyi networks, changing *a *has a strong effect on the dynamics. We then studied how the activating fraction affects the ability of a mutation/selection process to optimize the dynamical behavior. We numerically optimized 10-node networks to robustly reach steady state or oscillate, using the great deluge algorithm [28]. The convergence rate of the algorithm reflects the density of networks in parameter space: optimization converges more rapidly in regions of parameter space in which desirable networks are dense. We found that the number of mutations required to change a random graph into a network with robust dynamics strongly varies with *a*.

Although robustness is a key property of biological networks [11,29,30] there is no consistent definition of network robustness. The choice of definition is important because increasing one type of robustness can decrease another type [31]. We considered three types of robust dynamical behavior: *topological robustness *means the dynamics are robust to alterations in the network wiring; *mutational robustness *means the dynamics are robust to alterations in the interaction strengths (with no changes to which nodes are connected); and *environmental robustness *means that the dynamics are robust to changes in the initial conditions. We optimized for topologically and environmentally robust networks: we perturbed the network topology and measured how the dynamics changed, averaged over many initial conditions. To quantify robustness, we measured changes in the steady state (if a steady state is reached) or the oscillation period (see Methods).

**Figure 3:**
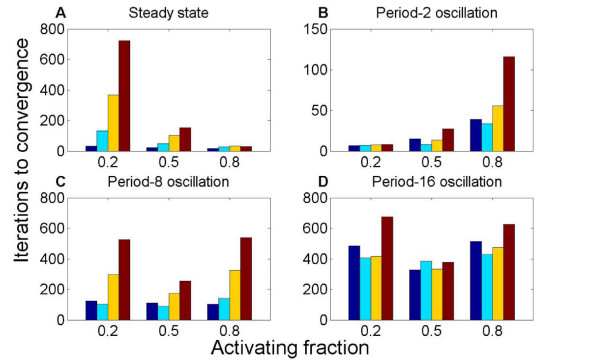
**The activating fraction *a *and optimization of dynamical properties.** The number of iterations to convergence of the optimization algorithm, as a function of *a*. 10-node networks were optimized to robustly reach steady state (A) or oscillate (B-D). Different colors represent different levels of connectedness: from left, the degree is 1 (blue), 2 (cyan), 5 (yellow), or 8 (red). Convergence to dynamics that (A) reach steady state or (B) oscillate with period 2 are most rapid for high or low *a*, respectively. Optimization for period-8 (C) and period-16 (D) oscillation converges most rapidly for intermediate *a*.

Networks which robustly reach steady state were rapidly found by our optimization for high *a *= 0.8: the optimization required 26 iterations to converge, on average (fig. 3a). For lower *a*, the results depend on the network degree: the increase in number of iterations to converge was a factor of ~2 to 25 when *a *was decreased to 0.2. Small increases occurred in *a *when selecting for steady state behavior. The networks that were initialized with the lowest value of *a *= 0.2 showed statistically significant increases in the average *a *of 0.13–0.45.

Optimization for low-period oscillation converged most rapidly for low *a *and for long-period oscillation at intermediate *a *(fig. 3b–d). We note that robust period-2 oscillation was found with fewer iterations of the optimization procedure than robust steady state, while long-period oscillation required many more iterations. For period-2 oscillation, the convergence slowed with *a *in a way that depended on the network degree.

When we optimized for long-period oscillation, a non-optimal *a *of 0.2 or 0.8 required 1.6–2 times more iterations to converge than an optimal *a *of 0.5, for a degree of 8 connections per node (fig. 3c–d). When selecting for long-period oscillation, we observed statistically significant (see Methods) shifts in *a *of the selected networks: the activating fraction shifted toward *a *= 0.5.

Both our network-optimization and random network results suggest that high *a *promotes dynamics that reach steady state, while intermediate and low *a *promotes oscillatory dynamics. Low *a *is correlated with short-period oscillation; intermediate *a *is correlated with long-period oscillation. To understand the connection between these observations and the dynamics of biochemical networks, we studied 5 relatively small, and therefore tractable, biological network topologies. While we did not attempt to replicate biochemically realistic dynamics of these networks, we sought to understand how the network topology and activating fraction together affect the dynamics. For biochemical networks, the network topology is clearly important, because these networks have been influenced by billions of years of evolution. But are such networks therefore insensitive to changes in *a*?

We studied two circadian oscillators and three signaling networks: the 10-node *Drosophila *circadian clock [32], the 8-node *Arabidopsis *circadian clock [33], the 15-node core Notch pathway [34], the 27-node Wnt/*β*-catenin signaling pathway [35]; and the 22-node Nerve Growth Factor (NGF) pathway [36] (see Methods for details). We expect circadian clock networks to oscillate, and the signaling networks to reach steady state. Consistent with this idea, the circadian networks have an average *a *of 0.74, while the signaling networks have a higher average *a *of 0.83.

We first studied the known topology of each network. On average, the circadian clock network dynamics oscillate in > 95% of trials, while the signaling network dynamics reach steady state in 80% of trials. This result suggests that our simple model captures features of the biological network dynamics. Because numerical values for the connection strengths are not known, we averaged over randomly chosen interaction strengths, drawn from a Gaussian distribution with unit mean and variance. Therefore, the biological networks we studied show high *mutational *and *environmental robustness *because their dynamics are not sensitive to the exact values of the interaction strengths or initial conditions. Two of the signaling networks' dynamics (Wnt and Notch) always reach steady state; the networks have no feedback loops, which are required for oscillations.

**Figure 4:**
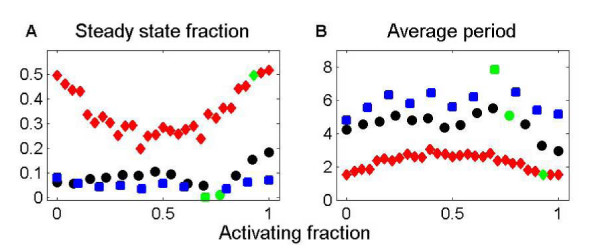
**The activating fraction *a *and the dynamics of biochemical networks.** The probability the dynamics reach steady state (A) and the average period of oscillation (B) as a function of *a*. The networks are the *Drosophila *circadian oscillator (black circle), the *Arabidopsis *circadian oscillator (blue square) and the NGF signaling network (red diamond). The point corresponding to the natural *a *is green. The NGF network dynamics typically reach steady state. The circadian network dynamics have a low probability of reaching steady state, but the probability is increased 20-fold when *a *is high.

We then varied *a *from its natural value by changing the sign of some interactions in the network, without changing which nodes are connected. For the circadian networks, the fraction of runs that reach steady state, while always low, increases dramatically for unnatural values of *a*. Increasing *a *to 1 by making all connections positive increases the chance of reaching steady state from ~1% to 18% for the *Drosophila *circadian network. For the *Arabidopsis *circadian network, this increase in *a *increases the chance of reaching steady state from 0 to 8% (fig. 4). The oscillation period for the circadian networks is longest near the natural *a*. Both the topology and the pattern of activating and inhibiting connections is important for circadian network dynamics.

The NGF network dynamics reach steady state much more often than do the circadian-network dynamics. For the natural *a*, ~50% of runs reach steady state. A decrease to *a *= 0.5 decreased the chance of reaching steady state to 20% (figure 4). The NGF network dynamics showed a non-monotonic dependence of the steady state fraction on *a*, for reasons not understood. The other two signaling networks have dynamics without feedback that reach steady state for all values of *a*.

We note that the results presented here normalize the baseline activation state of genes in a given organism to zero, and thus a change of the default state of genes from on to off would change the overall network properties, and hence the balance of activators to repressors. For example, in eukaryotes, most genes are turned off by default, and the ratio of positive to negative regulators in *S. cerevisiae *is about 3:1 in favor of positive regulations [37], whereas in bacteria, most genes are turned on by default, and the Regulon database [38] accordingly suggests that *E. coli *has a lower ratio, 3:2 in favor of positive regulators. Determining whether networks with different basal levels of expression but the same ratio of activators to repressors relative to baseline would be a fascinating topic for empirical studies, for example by changing the properties of the nodes of the repressilator.

## Conclusion

Our results suggest that the fraction of activating connections *a *is an important determinant of network dynamics. In the biochemical networks we studied, the specific network topology is clearly important. However, changing *a *also significantly altered the network dynamics. The circadian networks at their natural activating fraction almost always oscillate; increasing the activating fraction decreases the average oscillation period. The NGF signaling network dynamics become half as likely to reach steady state as *a *is changed from the natural value.

Much current research on biological networks focuses on finding network features that determine the behavior of the network, including network dynamics and the response of the network to stimuli or perturbations [39,40]. Our result that *a *strongly affects network dynamics has implications for the evolution of biological clocks and switching networks. An intermediate value of *a *= 0.5 increases both the chance of long-period oscillations (in random Erdös-Renyi networks) and rate of convergence to a network with long-period oscillation (in optimized networks). This suggests that a network with equal numbers of activiating and inhibiting connections may be at an advantage in evolving a circadian clock. When *a *is high, random network dynamics are likely to reach steady state and optimized networks that reach steady state are most rapidly selected. Biochemical networks which reach steady state are more likely to be robust when the fraction of activating connections is high.

## Methods

### Model

We adapted the model of Wagner [15], as used by Siegal and Bergman [18,19]. The network consists of nodes (representing genes, mRNA transcripts, or proteins) and edges (interactions between pairs of nodes). Each node potentially has an effect on each other node. This effect is represented by a number, *W*_*ij*_, which describes the strength of the effect of node *j *on node *i*. An interaction strength can be positive (activating connection), negative (inhibiting connection), or zero (no connection). The interactions among the *N *nodes are collected in an *N *× *N *matrix *W*. Note that nodes can regulate themselves; these terms are the *W*_*ii *_entries of the matrix.

Each network node has an activity level, which represents how activated or repressed the node is. The activity level could represent the expression (if the node is a gene or transcript) or activity level (if the node is a protein). The relative activity of node *i *is represented by *s*_*i*_, which lies between -1 and 1. An activity level of 0 represents the basal activity of the node, while an activity level of 1 or -1 describes a node which is maximally activated or maximally inhibited, respectively. The activity of each node changes in time due to the effects of other nodes. The activity levels of all *N *nodes are collected in an *N*-element vector **s**. The activity vector changes in time; the dynamical rule which describes this change is the most important feature of the model. Given an activity vector **s**(*t*) at a specific time *t*, the activity vector at the next time, **s**(*t *+ 1) must be determined. For node *i*, we sum over *j *the product of the activity level of node *j *times the interaction strength of node *j *on node *i*. This models the idea that node *j *has an effect on node *i *that depends both on the interaction strength and the activity of node *j*. Multiplying the interaction strength by the activity of node *j *produces the actual effect of *j *on *i *under current conditions. These effects are summed over all nodes that regulate *i*. We then transform the result by applying a nonlinear function *f*

(2) $$s_{i}(t+1)=f\left(\sum_{j=1}^{N}W_{ij}s_{j}(t)\right).$$

The quantity ∑*W*_*ij*_*s*_*j *_may lie outside the range (-1, 1), and so therefore may not be a valid activity level for node *i*. The nonlinear function enforces the saturation of interactions among nodes: this models the idea that no matter how strong the activating (repressing) influence on a node, there is a maximum (minimum) activity level that can be attained. We use the nonlinear function *f*(*x*) = tanh(*rx*) with *r *= 100.

The dynamical rule describing the time evolution of the interacting genes is given in eq. 1.

### Network dynamics

Starting from a random initial condition, we iterate the dynamics (eq. 1) to determine the activity vector as a function of time. We then compute the difference between a given **s**(*t*) and the expression vectors at the previous time steps, **s**(*t *- 1), **s**(*t *- 2), and so on. To determine whether a steady state has been reached, we examine whether the Hamming-like distance

(3) $$d(s,s^{\prime })=\sum_{i}{\left(s_{i}-{s^{\prime }}_{i}\right)}^{2}/(4N)=\frac{{\left(s-s^{\prime }\right)}^{2}}{4N}$$

between the vectors at time points *t *and *t *- 1 is below some threshold *δ*. Then the vectors are considered equal, and the dynamics have an oscillation with period 1, a steady state. (Typically we use the value *δ *= 10^-4^, although we verified that varying *δ *between 10^-2 ^and 10^-10^did not change the results.) To determine the period of oscillation, we examine whether the last *n *values of **s **are equal (within error) to the next-to-last *n *values for any value of *n*. Note that a steady state is equivalent to an oscillation with period *n *= 1, because at steady state we have **s**(*t*) = **s**(*t *- 1).

When determining the oscillation period, we iterate the dynamics a fixed number of times, typically 100. Therefore we cannot distinguish between chaotic dynamics and oscillations with period longer than 100.

### Network sampling

To develop a sample of networks with given properties, we specify the network size *N*, the probability of (nonzero) connection between nodes *c*, the fraction of activating connections *a*, and the distribution of connection strengths. Each characteristic is separately controlled. In this work we used a classical Erdös-Renyi random network model, where a connection between any two nodes is made with probability *c*. Therefore the average number of nodes that affect any given node in the network is *cN*. The activating fraction 0 ≤ *a *≤ 1 determines the probability that two connected nodes have a positive (activating) interaction strength. The remaining nonzero connections have a negative interaction strength, which corresponds to an inhibiting interaction. We varied these parameters to study networks with *N *between 10 and 100, connection probability between 0.01 and 1 and activating fraction between 0 and 1.

The absolute value of the connection strength is randomly chosen according to a specified distribution. We used a wrapped Gaussian distribution, where the values are chosen from a Gaussian distribution but any negative values are made positive. The mean *μ *and standard deviation *σ *of the Gaussian distribution were varied in our simulations. We used distributions with mean between 0 and 1 and standard deviation between 0.03 and 1. Altering parameters which control the magnitude of connection strengths has little effect on the dynamics, on average: neither the fraction of runs which reach steady state nor the average period is sensitive to alteration in the mean or standard deviation of the connection strength distribution. The connection strength parameters have little effect because the nonlinear function used in our dynamics is nearly always saturated.

We implemented the graphs using arrays in the Python Numeric [41] package. The graph topology and the edge weights were generated separately, allowing us to vary the topology and the connection strengths and signs independently.

### Robustness measures

We consider three different types of robustness: *topological robustness*, which means network dynamics are robust to alterations in the network wiring; *mutational robustness*, which means the network dynamics are robust to alterations in connection strength; and *environmental robustness*, which means that the network dynamics are robust to changes in the initial condition.

In our model, each type of robustness requires a different perturbation to the network and measure of the change in behavior. We implement the perturbations corresponding the three types of robustness as follows: (*i*) *Topological perturbation*: we alter the network by randomly deleting one connection and then randomly adding a connection between two previously unconnected nodes, with random strength. The value of the strength of the connection is drawn from the same distribution used to originally make the network. This model preserves the total number of connections in the network. (*ii*) *Mutational perturbation*: we randomly select one (nonzero) connection and alter its strength. The strength of the connections is drawn from the same distribution used to originally make the network. (*iii*) *Environmental perturbation*: we alter one node of the vector of initial conditions.

To quantify robustness, we use different measures, depending on whether the network dynamics reach steady state or oscillate with period > 1. The robustness measure for dynamics which reach steady state is the mean phenotypic distance [18]. This measure assumes that both the original and perturbed networks reach a steady-state activity vector. We determine the steady-state activity vector **s**_*r *_of a reference matrix and initial condition **s**_0_. Then we alter the matrix and/or initial condition *M *times, and determine a set of *M *steady-state vectors **s**_*p*_(*j*) that result from the perturbation (here *j *= 1, 2, ⋯ *M*). The mean phenotypic distance ${\bar{d}}_{p}$ is the average Hamming-like distance (eq. (3)) between each perturbed activity vector and the reference activity vector

(4) $$\bar{d_{p}}=\frac{1}{M}\sum_{j=1}^{M}\frac{{\left(s_{r}-s_{p}(j)\right)}^{2}}{4N}.$$

If the perturbations to the network tend to lead to the same steady state activity levels, the mean phenotypic distance will be low. In this case we say that the network is robust to the alteration. Typically we use *M *= 100.

For networks that oscillate, we measure robustness either using the mean absolute change in period or the mean change in period distribution. To determine the mean absolute change in period, we first determine the period of oscillation *p*_*r *_of a reference matrix and initial condition. We then perturb the matrix and/or initial condition *M *times, and determine a set of *M *oscillation periods *p*_*p*_(*j*) for each perturbed network (here *j *= 1, 2, ⋯ *M*). Then the mean absolute change in period is

(5) $$\bar{\left|\Delta_{p}\right|}=\frac{1}{M}\sum_{j=1}^{M}\left|p_{r}-p_{p}(j)\right|.$$

For a more detailed measure of robustness of oscillatory networks, we study the mean change in period distribution. This measure allows us to study the effects of both varying initial conditions and alterations in the interaction network, and assess both steady-state and oscillating network dynamics. We consider a reference network and a set of *M *perturbed networks. For each network, we sample *k *different initial conditions. Each initial condition leads to a period of oscillation (where period 1 represents steady state). For a given network, we determine the period distribution: the number of times that the different initial conditions lead to oscillations of period 1, period 2, and so on. The vectors of counts are **v**_*r *_for the reference matrix and **v**_*p*_(*j*) for the perturbed networks (here *j *= 1, 2, ⋯ *M*). The mean change in period distribution is

(6) $$\bar{\left|\Delta v\right|}=\frac{1}{M}\sum_{j=1}^{M}\sqrt{{\left(v_{r}-v_{p}(j)\right)}^{2}}.$$

If the alterations in the network or initial condition tend to lead to the same period of oscillation, the mean change in period distribution will be low. In this case we say that the network was robust to the alteration.

### Network optimization

We used the great deluge algorithm (GDA) [28] to test whether graphs with specific properties are more abundant in some regions of parameter space than others. At each step in the optimization, one edge of the graph was randomly changed to a new value, drawn from the same distribution used to create the graph (which was a Gaussian with unit variance "wrapped" by taking the absolute value of all values, so all draws have the same sign). The optimization was repeated for starting populations of graphs with different values of *a *and *c*, as shown in table 1.

**Table 1:** Network selection results

Period	*a*	*c*	⟨*a*⟩	Std error	Change in *a*	P value	Iterations to converge	Std error
1	0.2	0.1	0.227	1.6 × 10^-3^	13.61%	1.5 × 10^-06^	33.5	1.9
		0.2	0.255	2.6 × 10^-3^	27.41%	6.9 × 10^-30^	131.0	5.8
		0.5	0.270	6.3 × 10^-3^	35.21%	3.8 × 10^-32^	367.2	21.1
		0.8	0.290	7.8 × 10^-3^	45.06%	4.5 × 10^-33^	722.4	46.1

	0.5	0.1	0.525	1.4 × 10^-3^	5.07%	9.0 × 10^-06^	21.4	0.9
		0.2	0.533	1.8 × 10^-3^	6.64%	1.7 × 10^-14^	47.7	1.6
		0.5	0.517	2.9 × 10^-3^	3.43%	1.5 × 10^-06^	102.7	4.2
		0.8	0.518	3.3 × 10^-3^	3.62%	4.8 × 10^-07^	153.4	7.9

	0.8	0.1	0.823	1.3 × 10^-3^	2.83%	1.3 × 10^-07^	15.7	0.5
		0.2	0.828	1.7 × 10^-3^	3.47%	3.5 × 10^-18^	27.5	0.7
		0.5	0.818	2.3 × 10^-3^	2.31%	8.6 × 10^-18^	32.3	0.7
		0.8	0.813	1.8 × 10^-3^	1.65%	1.0 × 10^-14^	28.8	0.7

2	0.2	0.1	0.247	5.3 × 10^-3^	23.65%	0.064	6.5	1.2
		0.2	0.192	7.6 × 10^-3^	-3.92%	0.66	7.3	1.5
		0.5	0.207	9.4 × 10^-3^	3.50%	0.51	7.6	1.0
		0.8	0.199	8.7 × 10^-3^	-0.30%	0.94	8.0	1.3

	0.5	0.1	0.465	5.2 × 10^-3^	-7.01%	0.25	14.9	3.1
		0.2	0.505	7.3 × 10^-3^	1.01%	0.82	8.1	1.2
		0.5	0.497	9.7 × 10^-3^	-0.50%	0.86	13.5	1.8
		0.8	0.500	6.6 × 10^-3^	-0.07%	0.97	27.1	4.1

	0.8	0.1	0.752	5.2 × 10^-3^	-6.05%	0.0063	38.9	5.5
		0.2	0.778	8.5 × 10^-3^	-2.76%	0.1	33.5	6.4
		0.5	0.753	1.0 × 10^-3^	-5.93%	1.4 × 10^-06^	55.8	6.9
		0.8	0.767	8.6 × 10^-3^	-4.15%	9.7 × 10^-07^	115.8	13.5

8	0.2	0.1	0.229	4.4 × 10^-3^	14.74%	0.071	123.8	17.1
		0.2	0.231	5.5 × 10^-3^	15.59%	0.0013	103.7	7.3
		0.5	0.244	1.1 × 10^-3^	22.23%	9.3 × 10^-09^	296.2	25.9
		0.8	0.273	7.3 × 10^-3^	36.60%	3.2 × 10^-23^	527.0	47.6

	0.5	0.1	0.459	4.3 × 10^-3^	-8.15%	0.062	111.8	14.4
		0.2	0.486	6.5 × 10^-3^	-2.70%	0.42	90.5	7.8
		0.5	0.485	8.5 × 10^-3^	-3.00%	0.11	174.1	14.1
		0.8	0.498	4.7 × 10^-3^	-0.50%	0.60	253.7	11.6

	0.8	0.1	0.800	5.9 × 10^-3^	0.01%	0.99	102.3	10.9
		0.2	0.785	7.1 × 10^-3^	-1.84%	0.18	141.6	19.0
		0.5	0.747	8.6 × 10^-3^	-6.66%	5.4 × 10^-12^	325.3	2.04
		0.8	0.732	6.9 × 10^-3^	-8.53%	9.2 × 10^-15^	540.2	34.1

16	0.2	0.1	0.210	4.3 × 10^-3^	5.11%	0.47	483.9	52.8
		0.2	0.221	5.6 × 10^-3^	10.38%	0.017	406.1	32.7
		0.5	0.245	9.9 × 10^-3^	22.72%	4.4 × 10^-09^	416.9	36.1
		0.8	0.273	6.7 × 10^-3^	36.72%	1.6 × 10^-20^	676.9	44.9

	0.5	0.1	0.503	6.5 × 10^-3^	0.50%	0.89	327.9	29.6
		0.2	0.497	7.3 × 10^-3^	-0.56%	0.84	386.9	34.4
		0.5	0.501	7.6 × 10^-3^	0.16%	0.92	333.0	23.5
		0.8	0.502	4.6 × 10^-3^	0.46%	0.63	379.1	15.0

	0.8	0.1	0.754	4.4 × 10^-3^	-5.75%	0.0014	514.2	78.4
		0.2	0.793	4.8 × 10^-3^	-0.93%	0.42	429.9	37.6
		0.5	0.754	8.6 × 10^-3^	-5.74%	6.4 × 10^-08^	473.5	36.7
		0.8	0.739	8.2 × 10^-3^	-7.65%	3.6 × 10^-18^	625.0	36.3

Column 1: period optimized for. Column 2: activating fraction used to generate the network. Column 3: connection probability. Column 4: average activating fraction in optimized networks. Column 5: standard error of average activating fraction. Column 6: average change in activating fraction. Column 7: P value for the change in activating fraction. Column 8: number of iterations for the GDA to converge. Column 9: standard error of iterations to convergence.

Regions of parameter space in which desirable networks are dense converge in few iterations, whereas regions where they are sparse require more iterations for convergence. Therefore, the convergence rate of the algorithm discriminates between parameters where a desired dynamical behavior is common or uncommon. The GDA is a nonlinear optimization algorithm that performs well on a range of highly nonlinear problems and converges more rapidly than simulated annealing or genetic algorithms [28]. When used for maximization (seeking the largest value of a fitness function), the fitness surface is imagined as a landscape where higher values (peaks) are better. The GDA uses the concept of a "water level", below which changes are unacceptable. A random walk is performed on the landscape of possibilities, and the fitness is evaluated at each step. If the fitness at the new location is higher, the new location is accepted and the water level rises by a constant value based on the initial cost. (This value was selected after trial runs; we attempted to balance the convergence rate with the quality of the solution found.) If the fitness at the new location is lower but above the water level, the change is accepted. If the fitness at the new location is below the water level, the change is rejected.

The great deluge algorithm is less sensitive to local optima than is hill-climbing (always accepting the solution if it is better) or gradient methods, and is typically faster and less parameter-dependent than algorithms such as simulated annealing (for which temperature parameters and gradients must be selected) or genetic algorithms (for which a large population of solutions and a number of parameters are required). In our simulations, a random step in the fitness landscape was performed by a single perturbation to the network. The fitness function we used is the sum of the phenotypic distance and the mean change in period distribution, so $F=\bar{d_{p}}+\bar{\left|\Delta v\right|}$. An increase in fitness as a result of a perturbation means that the new graph is more robust (to both changes in the network wiring and changes in the initial condition) than the previously proposed graph. We measured the number of iterations required for the GDA to converge, and we discarded runs in which no convergence was obtained after 10,000 steps.

To test whether the connection probability and the activating fraction had changed, we compared the known means for the starting population to the sample means for the selected population using one-tailed, one-sample *t *tests (*n *was approximately 50 in each sample). Because each *t *test is an independent test of the hypothesis that the sample means had changed in the direction predicted, we used Fisher's method of combining independent tests of a hypothesis [42] to test whether the overall level of change was significant. We compared mean convergence times between each pair of parameter settings using two-sample *t *tests assuming unequal variances.

The results of the network selection are shown in detail in table 1.

### Biological network topologies

We studied five network topologies: the 10-node *Drosophila *circadian network sketched in reference [32], figures 4/6; the 8-node *Arabidopsis *circadian network sketched in reference [33], figure 4; the 15-node core Notch pathway sketched in reference [34]; the 27-node WNT/*β*-catenin signaling pathway sketched in reference [35]; and the 22-node Nerve Growth Factor (NGF) signaling pathway sketched in reference [36], figure 3. Details of the size and number of activating connections for these networks are given in table 2. The representation of network topologies in our model is given in tables 3, 4, 5, 6, 7.

**Table 2:** Activating connections in biological network topologies.

Network	Nodes	Connections	Activating connections	Activating fraction
*Drosophila *circadian clock	10	13	10	0.77
*Arabidopsis *circadian clock	8	10	7	0.70
Nerve Growth Factor signaling	22	28	26	0.93
WNT/*β*-catenin signaling	27	30	23	0.77
Notch signaling	15	15	12	0.8

This table summarizes characteristics of the topologies of several biological networks.

**Table 3:** Drosophila circadian clock.

Upstream node	Downstream node	Sign of interaction
Tim mRNA	Tim	+
Per mRNA	Per	+
Vri mRNA	Vri	+
Pdp1e mRNA	Pdp1e	+
Clk mRNA	Clk	+
Clk	Tim mRNA	+
Clk	Per mRNA	+
Clk	Vri mRNA	+
Clk	Pdp1e mRNA	+
Pdp1e	Clk mRNA	+
Per	Clk	-
Tim	Clk	-
Vri	Clk mRNA	-

This table shows the connections that comprise the topology of the *Drosophila *circadian network.

**Table 4:** Arabidopsis circadian clock.

Upstream node	Downstream node	Sign of interaction
X mRNA	X	+
X	Lhy mRNA	+
Lhy mRNA	Lhy	+
Toc1 mRNA	Toc1	+
Toc1	X mRNA	+
Y mRNA	Y	+
Y	Toc1 mRNA	+
Lhy	Y mRNA	-
Lhy	Toc1 mRNA	-
Toc1	Y mRNA	-

This table shows the connections that comprise the topology of the *Arabidopsis *circadian network.

**Table 5:** Notch signaling network.

Upstream node	Downstream node	Sign of interaction
Fringe	Notch	+
Dvl	Notch	-
Numb	Notch	-
Notch	Deltex	+
*γ*-Secretase complex	Notch	+
TACE	Notch	+
Serrate	Notch	-
Delta	Notch	+
Notch	CSL	+
SKIP	CSL	+
MAML/HATs	CSL	+
CSL	O DNA	+
Co-repressor complex	CSL	-
O DNA	Hes1/5	+
O DNA	PreT*α*	+

This table shows the connections that comprise the topology of the Notch signaling network.

**Table 6:** WNT/*β*-catenin signaling network.

Upstream node	Downstream node	Sign of interaction
WNT	WNT-Frizzled complex	+
DKK	WNT-Frizzled complex	-
WIF-1	WNT-Frizzled complex	-
WNT-Frizzled complex	pDSH	+
NAKED	pDSH	-
pDSH	PP2A	+
pDSH	pAXIN-pAPC-p*β*-catenin complex	-
CK-I/CK-II complex	pDSH	+
DSH	pDSH	+
PP2A	pAXIN	+
PP2A	p*β*-catenin	+
pAXIN-pAPC-p*β*-catenin complex	pAXIN	+
pAXIN-pAPC-p*β*-catenin complex	*β*-catenin	+
pAXIN-pAPC-p*β*-catenin complex	p*β*-catenin/*β*-TRCP complex	+
p*β*-catenin	p*β*-catenin/*β*-TRCP complex	+
*β*-TRCP	p*β*-catenin/*β*-TRCP complex	+
Ubiquitin pathway	p*β*-catenin/Ubiquitin complex	+
p*β*-catenin/*β*-TRCP complex	p*β*-catenin/Ubiquitin complex	+
p*β*-catenin/Ubiquitin complex	*β*-catenin fragments	+
Proteasome	*β*-catenin fragments	+
GSK3B	pAXIN-pAPC-p*β*-catenin complex	+
FRAT1	pAXIN-pAPC-p*β*-catenin complex	-
AXIN/APC/*β*-catenin complex	pAXIN-pAPC-p*β*-catenin complex	+
AXIN/APC/*β*-catenin complex	*β*-catenin/GROUCHO/Smad4 complex	+
*β*-catenin/GROUCHO/Smad4 complex	c-MYC/CYCLIN D1/PPAR *d *complex	+
GROUCHO complex	c-MYC/CYCLIN D1/PPAR *d *complex	-
HDAC	GROUCHO complex	+
GROUCHO complex	*β*-catenin/GROUCHO/Smad4 complex	+
TAK1/TAB1 complex	NLK	+
NLK	*β*-catenin/GROUCHO/Smad4 complex	-

This table shows the connections that comprise the topology of the WNT/*β*-catenin signaling network.

**Table 7:** NGF signaling network.

Upstream node	Downstream node	Sign of interaction
Ras	Raf	+
Raf	MEK1/2	+
MEK1/2	Erk1/Erk2	+
Erk1/Erk2	Rsk	+
Rsk	CREB	+
CREB	FasL/Bcl-2/Bax/Egr-1 mRNA	+
p38	FasL/Bcl-2/Bax/Egr-1 mRNA	+
Akt1/2	FasL/Bcl-2/Bax/Egr-1 mRNA	+
Forkhead	FasL/Bcl-2/Bax/Egr-1 mRNA	+
NF*κ*B	FasL/Bcl-2/Bax/Egr-1 mRNA	+
Trk receptor	PLC*γ*	+
PLC*γ*	IP3/DAG complex	+
IP3/DAG complex	PKC*δ*	+
PKC*δ*	MEK1/2	+
Frs2/Crk/DOCK-180/SH2B/Grb2 complex	Rac	+
Trk receptor	IRS-1	+
IRS-1	Shc/Grb2/SOS/Gab/PI3k complex	+
Shc/Grb2/SOS/Gab/PI3k complex	1	+
Shc/Grb2/SOS/Gab/PI3k complex	Akt1/2	+
PDKs	Akt1/2	+
Akt1/2	p38	-
Akt1/2	Forkhead	-
Akt1/2	NF*κ*B	+
Akt1/2	BAD/Bcl-2 complex	+
BAD/Bcl-2 complex	Bad/14-3-3 complex	+
Ras	Shc/Grb2/SOS/Gab/PI3k complex	+
Shc/Grb2/SOS/Gab/PI3k complex	MEK1/2	+
Trk receptor	Frs2/Crk/DOCK-180/SH2B/Grb2 complex	+

This table shows the connections that comprise the topology of the NGF signaling network.

In our simulations, we generated networks with topology corresponding to the biological network. Keeping this topology constant, we varied *A*, the number of activating connections, from 0 (producing a network that contains no activating connections, i.e., all connections are inhibiting connections) to *A*_*m*_, the number of edges in that network (all connections are activating connections). Since the network has *A*_*m *_edges, the activating fraction is *a *= *A*/*A*_*m*_. Note that we did not change which network nodes were connected, only the sign of the interaction (activating or inhibiting). We chose to pick the inhibiting connections to match the network to the biological network as much as possible. Therefore, as the number of inhibiting connections was increased, we first randomly selected from the inhibiting connections that occur in the real biological network, then (once all of those inhibiting connections were made) we randomly selected from the remaining connections in the network. For each choice of the number of inhibiting connections, we generated *k *= 10^3 ^networks with randomly chosen interaction strengths and random initial conditions. The signs of the connections of each of these networks were chosen independently. For example, if there were originally 5 inhibiting connections, each of the *k *= 10^3 ^networks generated for 3 inhibiting connections was independently assigned one of the $\left(\begin{array}{c} 5\\ 3 \end{array}\right)$ = 10 ways of choosing which edges remained inhibiting. For each of the *k *graphs and initial conditions we determined the period of oscillation. Summing over all *k *networks and initial conditions gave us a period distribution for the graph with *A *activating connections. The mean of this distribution is the average period for the network with *A *activating connections.

## Competing interests

The authors declare that they have no competing interests.

## Authors' contributions

DM wrote the code, ran simulations and interpreted results. LW ran preliminary simulations, participated in programming and interpreted results. RK directed the coding, participated in programming, and interpreted results. MB directed the project, analyzed results, and wrote the manuscript. All authors read and approved the final manuscript.

## Reviewers' comments

### Reviewer's report 1

Reviewer 1: Sergei Maslov, Department of Condensed Matter Physics and Materials Science, Brookhaven National Laboratory

**Reviewer's comment: **The manuscript reports an interesting study of how the balance between positive and negative regulatory interactions affects the dynamics of regulatory networks. This subject definitely deserves a careful analysis and (to the best of my knowledge) was not explored before. Authors use a simple dynamical model introduced by Andreas Wagner in 1996 and later used in a number of publications. The most appealing property of this model is its simplicity: the genes (or their protein products) exist in either "active", "on" state (+1) or "inactive", "off" state (-1). While this boolean property is somewhat relaxed in this manuscript, it still (approximately) applies to the majority of nodes. The dynamical rules of the model remind the threshold activation rules in model neural networks, where contributions of multiple inputs weighted by their connection strength *W*_*ij *_are simply added up and compared with the activation threshold of a node. As such they cannot model an arbitrary logical function of input variables (one of the many simplifications of the model).

My main concern about this model is that it represents inactive genes/proteins by -1 (instead of simply 0). As a consequence even inactive genes keep sending negative/positive signals to their targets which they are positively/negatively regulating. This property of the model is rather artificial since in most cases genes/proteins that are not active *send no signal* instead of sending a *negative* signal. This can be easily rectified by the simultaneous change of *s*_*i *_variables (*s*_*i *_→ (*s*_*i *_+ 1)/2) and activation thresholds (equal to 0 in the present model). However, as a result of this transformation the activation thresholds become non-trivially (and unrealistically) coupled to magnitudes and *signs* of regulatory interactions *W*_*ij*_. As a consequence, I am not sure which of this study's conclusions about positive-to-negative ratio would survive in the more realistic scenario of arbitrary activation thresholds.

**Authors' response: **This simplification is noted by Wagner [15]: "For reasons of computational simplicity... ", and, as the reviewer notes, has been widely used in subsequent work (e.g. Siegal and Bergman 2002 [18]). It is precisely for the reason that the reviewer notes (that a large number of arbitrary activation thresholds must be introduced) that the variable transformation to a scale of 0 – 1 is not typically used. We would argue that our results show that in a widely used model of gene expression, we are able to identify key parameters related to network dynamics and to relate them to biological networks: there are many other restrictions of this admittedly simplified model that could be relaxed, and exploring which of these features is most influential for the results would be a fascinating topic for future work.

**Reviewer's comment: **Another simplification of the model is the synchronous update rule in which the states of all nodes are modified simultaneously with each other. In reality regulatory networks are characterized by a broad distribution of timescales of gene activation/deactivation corresponding to a more complicated (and generally asynchronous) update rule. Can authors comment on how sensitive are their results with respect to update rules?

**Authors' response: **Wagner (1996) also noted this limitation and cited the need for computational simplicity again. We did not model different update rules in this study, so we are unable to comment on the sensitivity. The simplicity of this model is sufficient to capture basic dynamics of a network and the model is further supported by its ability to represent "biological networks" as well as the repressilator. The total runtime was over 100,000 CPU hours, and the work itself is algorithmically complex. The bottleneck is thus not the hardware, and the simplifications required in 1996 are still needed today despite Moore's Law. However, again we agree that testing which model assumptions affect the result will ultimately be important for establishing the generality of the work.

**Reviewer's comment: **My final remark on this study is that in its present form it does not reveal which network property is being optimized by the empirically observed ratio of positive and negative regulations. Perhaps, authors could use the following observation: the ratio between positive and negative regulations in the genome-wide regulatory network of baker's yeast appears to be around 3:1 in favor of positive regulations (S. Maslov, K. Sneppen, Physical Biology, 2005). This is consistent with the ratio authors observed in their examples of real-life small subnetworks/pathways (except for the NGF signaling network). On the other hand, according to the data from the Regulon database, the regulatory network in *E. coli *has fewer positive regulations (the empirical ratio is only 3:2 in favor of positive regulations). This could be due to the fact that the default state of most bacterial genes is "on". That is to say, they are being expressed at a considerable rate even in the absence of any positive regulatory signals. Hence, they require fewer positive and more negative inputs than comparable networks in eukaryotes such as S. cerevisiae whose default state is typically off. Could authors comment on what are their assumptions about the default state of genes and how the change of the default state would affect the results?

**Authors' response: **This is a very interesting point, and we have incorporated this suggestion into the discussion as shown below. We thank the reviewer for the suggested references.

"We note that the results presented here normalize the baseline activation state of genes in a given organism to zero, and thus a change of the default state of genes from on to off would change the overall network properties, and hence the balance of activators to repressors. For example, in eukaryotes, most genes are turned off by default, and the ratio of positive to negative regulators in *S. cerevisiae *is about 3:1 in favor of positive regulations [37], whereas in bacteria, most genes are turned on by default, and the Regulon database [38] accordingly suggests that *E. coli *has a lower ratio, 3:2 in favor of positive regulators. Determining whether networks with different basal levels of expression but the same ratio of activators to repressors relative to baseline would be a fascinating topic for empirical studies, for example by changing the properties of the nodes of the repressilator."

### Reviewer's report 2

Reviewer 2: Eugene V. Koonin, National Center for Biotechnology Information

**Reviewer's comment: **The paper uses simulations to explore network dynamics and reaches a clear and interesting conclusion: that the fraction of activating (as opposed to inhibitory) connections determines the dynamical regime, that is whether the network arrives at a steady state of goes into an oscillating regime. As interesting as these findings are, I am somewhat uncomfortable with the conclusion: "The activating fraction may be a control parameter that cells use to predispose a network to oscillate or reach steady state." I am not sure that this (seemingly) adaptationist interpretation is justified or that the uncovered principle is as general as implied here. In particular, the oscillatory regime certainly is natural in the case of the circadian clock but its wider relevance remains to be proven.

**Authors' response: **We agree with the reviewer that the adaptationist language may be too strong here, and have changed the sentence to read "The activating fraction may predispose a network to oscillate or reach steady state, and neutral evolution or selection of this parameter may affect the behavior of biological networks."

### Reviewer's report 3

Reviewer 3: Yu (Brandon) Xia, Bioinformatics Program and Department of Chemistry, Boston University (nominated by Mark Gerstein, Department of Molecular Biology and Biophysics, Yale University)

**Reviewer's comment:**1. You correlated the simulated dynamics with biological function for five biological networks. I am wondering how much of the correlation can be teased out by simple static topological measures, such as number and percentage of feedback loops, and fraction of activation links?

**Authors' response: **We believe that the value of the present work is that we are able to show a link between even these static topological properties and the dynamics. Explaining the fraction of the variance in the behavior of real biological networks that is explained by these measures, as opposed to other properties, awaits both the development of more realistic models and the measurement of network dynamics in far more detail. However, it will be fascinating to explore these relationships in future.

**Reviewer's comment:**2. It will useful to illustrate some of these networks. For example, it will be useful to have one figure for a random network, one figure for an optimized network, and one figure for an actual biological network. It will be easier for the readers to see the similarities and differences between these networks.

**Authors' response: **We considered adding a few example networks, but in the end decided that it would be better to provide a recipe that allows interested readers to generate these networks with any arbitrary parameter settings, including those used throughout the paper. Accordingly, the following code snippet can be used to generate and display such networks:

>>>from networkx import XDiGraph, draw

>>>from numpy.random import normal

>>>from random import random

>>>from pylab import show

>>>def f(n, p):

...   g = XDiGraph( )

...   g.add_nodes_from(zip(range(n), [choice([-1,1]) for i in range(n)]))

...   for n1 in g.nodes( ):

...      for n2 in g.nodes( ):

...          if random( ) < p:

...            g.add_edge(n1, n2, normal(0,1))

...   return g

...

>>>g = f(20,0.1) #this is just an example: any value of n and p can be used

>>>draw(g)

>>>show( )

This can be used to generate random graphs equivalent to those used in the study. The dependencies are the NetworkX, numpy, and matplotlib packages.
